# Age-related differences in interference control in the context of a finger-lifting task: an fMRI study

**DOI:** 10.1093/scan/nsad034

**Published:** 2023-06-06

**Authors:** Federica Riva, Ekaterina Pronizius, Melanie Lenger, Martin Kronbichler, Giorgia Silani, Claus Lamm

**Affiliations:** Social, Cognitive and Affective Neuroscience Unit, Department of Cognition, Emotion, and Methods in Psychology, Faculty of Psychology, University of Vienna, Vienna 1010, Austria; Social, Cognitive and Affective Neuroscience Unit, Department of Cognition, Emotion, and Methods in Psychology, Faculty of Psychology, University of Vienna, Vienna 1010, Austria; Centre for Cognitive Neuroscience, University of Salzburg, Salzburg 5020, Austria; Centre for Cognitive Neuroscience, University of Salzburg, Salzburg 5020, Austria; Neuroscience Institute, Christian Doppler Clinic, Paracelsus Medical University, Salzburg 5020, Austria; Department of Clinical and Health Psychology, Faculty of Psychology, University of Vienna, Vienna 1010, Austria; Social, Cognitive and Affective Neuroscience Unit, Department of Cognition, Emotion, and Methods in Psychology, Faculty of Psychology, University of Vienna, Vienna 1010, Austria

**Keywords:** imitation inhibition, aging, adolescence, temporoparietal junction, fMRI, interference control

## Abstract

Humans tend to automatically imitate others and their actions while also being able to control such imitative tendencies. Interference control, necessary to suppress own imitative tendencies, develops rapidly in childhood and adolescence, plateaus in adulthood and slowly declines with advancing age. It remains to be shown though which neural processes underpin these differences across the lifespan. In a cross-sectional functional magnetic resonance imaging study with three age groups (adolescents (ADs) 14–17 years, young adults (YAs) 21–31, older adults (OAs) 56–76, *N* = 91 healthy female participants), we investigated the behavioral and neural correlates of interference control in the context of automatic imitation using the finger-lifting task. ADs showed the most efficient interference control, while no significant differences emerged between YAs and OAs, despite OAs showing longer reaction times. On the neural level, all age groups showed engagement of the right temporoparietal junction, right supramarginal gyrus and bilateral insula, aligning well with studies previously using this task. However, our analyses did not reveal any age-related differences in brain activation, neither in these nor in other areas. This suggests that ADs might have a more efficient use of the engaged brain networks and, on the other hand, OAs’ capacity for interference control and the associated brain functions might be largely preserved.

## Introduction

Humans, among other animals (e.g. dogs, Range *et al.*, 2011), tend to involuntarily imitate each other (Heyes, 2011). This propensity to imitate others’ movements (Genschow *et al.*, 2019), gestures and postures (Scheflen, 1964; Stürmer *et al.*, 2000), speech patterns (Babel, 2011; Christiner and Reiterer, 2013; Virhia *et al.*, 2019) or even physically impossible movements (Liepelt and Brass, 2010) is called automatic imitation and has been the subject of extensive research over the last 20 years (for review, see Heyes, 2011; Cracco *et al.*, 2018; Darda and Ramsey, 2019). Yet, if we were imitating every movement we observe, this would adversely affect our daily social interactions (Cross and Iacoboni, 2014; Bischoff *et al.*, 2020). This is why we also need the ability to control automatic imitation tendencies.

Different stages of life, such as adolescence (Shaw *et al.*, 2008; for review, see Blakemore, 2008; Blakemore, 2012; Larsen and Luna, 2018) and older age (Chen *et al.*, 2014; Ferguson *et al.*, 2021; Kupis *et al.*, 2021; for review, see Moran, 2013), are characterized by significant changes in socio-cognitive functioning as well as by associated changes in brain structure and function (for review, see Gogtay *et al.*, 2004; Steinbeis, 2016; Frangou *et al.*, 2022). As such, investigating differences in control of automatic imitation across different stages of life seems of crucial importance and demands attention. Thus, the main aim of the current study was to investigate cross-sectional differences in the control of automatic imitation in three age groups [adolescents (ADs), young adults (YAs), and older adults (OAs)] on the behavioral as well as the neural level.

One of the most common tasks historically adopted to investigate the control of automatic imitation is the finger-lifting task developed by Brass *et al.* (2000). This task belongs to a class of stimulus-response compatibility (SRC) tasks and assesses control or inhibition of automatic imitation via the interference effect between incongruent and congruent trials, with higher values indicating a supposedly reduced control of automatic imitation (Heyes, 2011; Genschow *et al.*, 2017; Cracco *et al.*, 2018).

However, after the data for the present study had been collected, new evidence revealed that in the classical SRC paradigms (i.e. the one used here based on the original work of Brass *et al.*, 2000), imitation interference is undistinguishable from spatial interference, where a task-irrelevant spatial match or mismatch between stimulus and response influences task performance (Aicken *et al.*, 2007; Czekóová *et al.*, 2021). Moreover, spatial interference effects appeared to be larger in magnitude than imitative interference effects (Catmur and Heyes, 2011), blurring any inferences about underlying imitation control when not controlled for using a dedicated version of the task. Besides that, the results of a recent well-powered MRI study provided no evidence for the validity and domain specificity of the SRC paradigms with regard to the social domain of automatic imitation (Darda *et al.*, 2018; for review, see Ramsey, 2018; cf. Cracco and Brass, 2019). Thus, while our original aim had been to investigate age-related differences in the inhibition of automatic imitation, we incorporated these new insights into reporting the results of our research. Our findings and approach will thus be framed as age-related differences in interference control more generally, and we will refrain from drawing any conclusions about possible underlying mechanisms specific to the social domain.

Interference control—the ability to suppress irrelevant responses—develops rapidly in childhood and adolescence, plateaus in adulthood and slowly declines with increasing age (Bedard *et al.*, 2002; Mayas *et al.*, 2012; for reviews, see Bessette *et al.*, 2020; Lustig and Jantz, 2015). According to the inhibition-deficit hypothesis (Hasher and Zacks, 1988), OAs struggle to suppress task-irrelevant overlearned responses or ignore distracting stimuli. On the other hand, studies focusing on age-related differences in interference control in the context of automatic imitation in adolescence and across the adult lifespan are scarce (for review, see Rauchbauer and Grosbras, 2020). To the best of our knowledge, only a single behavioral study explored interference control in the context of automatic imitation using the finger-lifting task across the adult lifespan and found that it decreases with age (Wermelinger *et al.*, 2018).

Therefore, the current study investigated age-related group differences in interference control at both the behavioral and neural level. We hypothesized a better performance, and thus a better interference control, in YAs compared to ADs and OAs. This has been motivated by prior studies on interference control (Bedard *et al.*, 2002; Mayas *et al.*, 2012) and by the findings of our group showing worse performance of ADs and OAs in a task investigating interference control in the emotional domain (Riva *et al.*, 2016).

At the brain level, neuroimaging studies in YAs (Brass *et al.*, 2009; Spengler *et al.*, 2009; Klapper *et al.*, 2014; Sowden and Catmur, 2015) using similar task versions found that the interference control is underpinned by the right temporoparietal junction (rTPJ) and the medial prefrontal cortex (mPFC). In prior work (Brass *et al.*, 2005, 2009), activation of rTPJ and mPFC in this task was associated with the involvement of theory of mind social networks (Gallagher and Frith, 2003; Schurz *et al.*, 2020). Note, though, that these regions are also involved in other, non-social processes (e.g. rTPJ and attention; Decety and Lamm, 2007; Schurz *et al.*, 2017; Schuwerk *et al.*, 2017). Recently, better-powered fMRI studies showed that interference control in this task engages rather domain-general brain areas, such as the dorsolateral prefrontal cortex, the inferior parietal lobule (IPL), and the inferior frontal gyrus (IFG), whereas the theory of mind network was found to be not active (Darda *et al.*, 2018; for a meta-analysis, see Darda and Ramsey, 2019).

Notably, both prefrontal cortical areas and cortical areas at the intersection of the posterior temporal and inferior parietal lobe, including not only the rTPJ but also the right supramarginal gyrus (rSMG), show a comparable structural developmental trajectory (with delayed full maturation in young adulthood and earlier decline as other areas; Gogtay *et al.*, 2004; Shaw *et al.*, 2008; Riva *et al.*, 2018; Natu *et al.*, 2019; Riva et al., 2022). Therefore, based on these data and the original findings of Brass *et al.*, (2009), we hypothesized that rTPJ and mPFC would underpin age-related behavioral differences in interference control in the context of a finger-lifting task. Given the publication of the above-mentioned meta-analysis by Darda and Ramsey (2019) after we had formulated the initial hypotheses and collected the data, we additionally explored (from now on called explorative analysis) age-related differences in brain areas that emerged in their study, namely, the rSMG, the right insula (rIns) and the left insula (lIns).

## Materials and methods

The present study was part of a larger project investigating socio-cognitive processes in different age groups, i.e. empathy, interference control in the emotional domain, as well as interference control in the automatic imitation inhibition context. This project imposed specific inclusion and exclusion criteria, including female gender, healthy neurotypical aging and specific age groups. Within the same experimental session (SM1.1 in the Supplementary material for further details), participants completed three tasks inside the MRI scanner: an empathy task (Lamm *et al.*, 2015), the emotional egocentricity bias task (Silani *et al.*, 2013) and the finger-lifting task (Brass *et al.*, 2000). The results are reported in Riva *et al.*, (2018), (2022) and in this paper.

### Participants

Ninety-six females were enrolled in this project. From these 96 participants, five had to be excluded for the following reasons: not complying with the instructions (*n* = 1), technical issues (*n* = 2) or excessive movements during scanning (*n* = 2). The final sample consisted, therefore, of 91 right-handed (Oldfield, 1971) female participants with normal or corrected-to-normal vision and with no history of psychiatric or neurological disorders (self-reported), divided into three age groups: ADs (*n* = 33, age range: 14–17), YAs (*n* = 29, 21–31 years) and OAs (*n* = 29, 56–76 years), following Riva *et al.*, (2016). Results of the post hoc power considerations are reported in SM1.2 in the Supplementary material and suggest that our study had sufficient power (0.8) for detecting upper medium to large effect sizes. OAs were tested using the German version of the Mini Mental State Examination (Kessler *et al.*, 2000; cut-off 27/30 as in Kukull *et al.*, 1994), and none of them showed the presence of early-stage neurodegenerative deficits. The demographic characteristics of the sample are reported in Table 1. The participants signed informed consent and received a monetary compensation of 25 euro for their participation. For ADs, we also obtained written informed parental consent. The study was approved by the ethics committee of the Medical University of Salzburg and was performed in accordance with the Declaration of Helsinki.

**Table 1.** Demographics

	Group		
**Measure**	**AD**	**YA**	**OA**
n	33	29	29
Age in years (SD)	15.58 (1.00)	24.52 (2.35)	63.41 (4.44)
Education in years (SD)	9.94 (1.08)	15.83 (3.02)	12.86 (3.31)

SD = standard deviation.

### The finger-lifting task

In the finger-lifting task (Brass *et al.*, 2000), the participants had to lift their index or middle finger in response to a cue, a number, that appeared on display (1 = index finger lift and 2 = middle finger lift). The cue was displayed on a sequence of pictures showing a human hand mirroring the participant’s hand. The first frame showed a still hand, while the second frame showed a hand lifting the same finger in the congruent condition, or the other finger, in the incongruent condition. Participants were instructed to ignore the movement of the observed hand, as it was irrelevant to the task, and to lift the finger indicated by the cue. The task consisted of randomly presented trials of 50 incongruent (25 index finger and 25 middle finger lift) and 50 congruent (25 index finger and 25 middle finger lift). The trials were distributed over the three blocks with short breaks between the blocks. The stimuli were presented using the software Presentation® (Version 18.0, Neurobehavioral Systems, Inc., Berkeley, CA, www.neurobs.com).

At the beginning of a trial, participants placed their index and middle fingers on two of the buttons of a response box. Each trial started with a fixation cross with a jittered time duration (4000 ms ± 2000 ms with steps of 500 ms), followed by a frame of a hand in the initial position (both fingers down) displayed for 1251 ms. Then, the three frames (34 ms + 34 ms + 1232 ms, in succession) depicted finger movements and a number cue (in congruent/incongruent trials). When the participant lifted their finger, the button on which the finger was positioned was relieved and the reaction time (RT) was recorded. The total task duration was 15 min.

### Neuroimaging data acquisition and preprocessing

Functional and structural MRI data acquisition was carried out on a 3 T Siemens Magnetom Trio scanner equipped with a 32-channel head coil. To obtain the structural scans, we used a sagittal T1-weighted magnetization-prepared rapid gradient echo sequence with the following settings: echo time = 2.91 ms, repetition time = 2300 ms, voxel size = 1 mm × 1 mm × 1.2 mm, slice thickness = 1.2 mm, field of view = 356 mm × 356 mm^2^, 192 slices and flip angle = 9°. To obtain the functional scans, we used a T2*-weighted echoplanar imaging (EPI) sequence with the following settings: 33 transverse slices covering the whole brain, echo time = 30 ms, repetition time = 2060 ms, slice thickness = 3 mm, field of view = 192 × 192 mm^2^, interslice gap = 0.3 mm, flip angle = 70° and matrix size = 64 × 64.

MRI data preprocessing and the following analyses were performed using SPM12 software (Statistical Parametric Mapping, Wellcome Trust Centre for Neuroimaging, http://www.fil.ion.ucl.ac.uk/spm) on MATLAB Version R2013a. Preprocessing steps included slice timing, realignment, co-registration of the EPI scans to the skull-stripped T1-weighted structural scan, spatial normalization (into Montreal Neurological Institute (MNI) space) and spatial smoothing with a 6-mm full-width at half-maximum (FWHM) Gaussian kernel. The brain regions were labeled using the SPM Anatomy toolbox version 2.15 (Eickhoff *et al.*, 2005) and the MRIcro atlas (aal.nii.gz).

### Analyses

#### Behavioral analysis

All statistical analyses of the behavioral data were performed using the IBM SPSS Statistics software (version 27, Released 2020, IBM Corp., Armonk, NY, USA).

We analyzed RTs and accuracy (percentage of correct reactions). For the RT data analysis, we removed trials with inaccurate finger lifts. To verify the existence of age-related differences in RTs and accuracy, a repeated measures analysis of variance (rm-ANOVA) including one within-subject factor Condition (2 levels: congruent and incongruent) and one between-subjects factor Group (3 levels: AD, YA and OA) was computed. The factor Finger was not included in the rm-ANOVAs; therefore, the two fingers were considered jointly. This was motivated by a preliminary analysis in which no relevant differences emerged due to the finger (see SM1.3 in the Supplementary material for details).

##### Percentage congruency effect.

For each participant, we calculated an interference effect controlled for age-dependent RT differences (PCE, equation (1), Forbes *et al.*, 2017),


(1) $$\begin{array}{*{20}{c}}
{PC{E_i} = \,\frac{{mean\,{{\left( {R{T_{incongruent}}} \right)}_i} - mean\,{{\left( {R{T_{congruent}}} \right)}_i}\,}}{{overall\,mean\,RT}}\,\times\,100\,}
\end{array}\\[8.5pt]$$

where *i* = participant *i* and overall mean RT = mean (RT_incongruent_ + RT_congruent_) across all participants (*N = *91).

The PCE indicates how much faster an individual participant’s RTs were for congruent compared to incongruent trials, relative to their overall mean RT (in %). The PCE score was used in a subsequent region of interest (ROI) analysis and a one-way ANOVA with a between-subjects factor Group (3 levels).

While we had initially intended to collect data also from an intermediate age group (age range between 40 and 50 years), we did not succeed in recruiting sufficient numbers of participants from this group. While this may be seen as suboptimal for some analyses, the way we dealt with this (as in our companion paper, Riva *et al.*, 2022) is to conduct and report different types of analyses (multiverse approach, see, e.g. Silberzahn *et al.*, 2018; Botvinik-Nezer *et al.*, 2020), which showed convergence of findings in large parts. To complement our categorical analysis, we calculated a multivariate linear regression in which congruent and incongruent RTs, as well as the PCE, were simultaneously entered as dependent variables and age as a continuous predictor (see SM2.1 in the Supplementary material for the results of the RT analyses and their interpretation, as well as the additional results of the curve estimation procedure, SM2.2 in the Supplementary material).

#### fMRI analyses

First-level, single-subject analysis was performed by adopting a general linear model approach (Friston *et al.*, 1995) as implemented in SPM12. Three contrasts of interest, congruent, incongruent and interference (incongruent—congruent) were computed for each subject and used for group-level analysis. Additionally, we applied spatial smoothing with a 6 -mm FWHM Gaussian kernel on the contrasts of interest (first-level, single participants) to increase the signal-to-noise ratio. For details on the regressors and timing, see SM1.4 in the Supplementary material.

Group-level analysis was performed following a three-step plan: first, we checked whether we replicated previous studies, i.e. Brass *et al.* (2009) (planned analysis) and Darda and Ramsey (2019) (explorative analysis) in YA in order to validate our study (results of the task validation of the whole sample can be found in SM2.3 in the Supplementary material); second, we conducted ROI analyses to test our hypotheses on age-related differences, and lastly, we complemented the ROIs analyses with a whole-brain analysis to explore possible age-related differences for which we had no specific hypothesis (beyond the planned and explorative ROIs).

For the first step, we conducted a one-sample *t*-test for the YA group, testing for significant group-level activation for the interference contrast on a whole-brain level. The initial (cluster-level selection) threshold was set at *P* = 0.001 uncorrected. To correct for multiple comparisons, we calculated the cluster extent threshold using ‘CorrClusTh.m’, an SPM extension script (Nichols and Wilke, 2012). We then computed two ROI analyses as a more explicit manipulation check testing for activation in previously identified brain areas (planned analysis, Brass *et al.*, 2009, explorative analysis Darda and Ramsey, 2019, Table 2). For the planned analysis, we built 9-mm radius spheres around the peak coordinates in rTPJ and mPFC based on the reported coordinates (Brass *et al.*, 2009). For the explorative analysis, we used the meta-analysis’s clusters of activation that survived the stringent extent-based thresholding and consisted of rTPJ, rSMG, rIns and lIns (Table 2). For further details on the ROIs definition and construction, see SM1.5 in the Supplementary material. Our manipulation check was successful for all but one ROI. We found no activation in mPFC for the interference contrast and, consequently, excluded it from further analyses. We have also tested brain–behavioral relationships, computing four correlations, one for each ROI, between the PCE scores and the mean ROI activity elicited by the interference effect controlled for age. The α level was corrected using the Bonferroni adjustment method to account for multiple comparisons in the explorative correlation analysis, where we had three ROIs (α/3 = 0.017).

**Table 2.** ROIs

Anatomical region	Hemisphere	MNI coordinates *x*, *y*, *z* in mm
mPFC^a,c^	L	2, 41, 20
TPJ^a^	R	52, −56, 20
SMG^b^	R	56, −36, 35
Insula^b^	R	36, 20, 3
Insula^b^	L	−35, 15, 0

*Note*: R = right, L = left.

a ROI derived from the study by Brass *et al.*, (2009).

b ROI derived from the meta-analysis by Darda and Ramsey (2019). The general compatibility map Activation_FWE_extent_stringent was taken from https://neurovault.org/collections/5377/.

c Since we found no activation in mPFC for the interference contrast, we excluded this ROI from further analyses.

The second step consisted of testing age-related differences in interference control. To this aim, we extracted mean activation of the interference contrast of all participants of the three age groups within the ROIs from Brass *et al.*, (2009) (planned analysis) and Darda and Ramsey (2019) (explorative analysis). To test for group differences in these ROIs (rTPJ, rSMG, rIns and lIns, Table 2), we computed four one-way ANOVAs, one for each ROI, with Group (3 levels: AD, YA and OA) as a between-group factor. Next, we computed correlations between the ROIs extracted values and age, controlled for multiple comparisons. To investigate the mismatch between the behavioral results, with YA and OA performing significantly worse than AD, and the brain results, showing no group differences in brain activity, we also specified a multiple regression model that looked at associations between behavioral and brain data (see SM2.4 in the Supplementary material for details).

For our third step, the complementary whole-brain analysis investigating potential age-related differences across the whole brain, we computed a flexible factorial design with participants as a within-participants factor, Group as a between-group factor (AD, YA and OA) and Interference as a within-group factor (incongruent—congruent). Our four contrasts of interests were AD > YA, AD < YA, OA > YA and OA < YA.

## Results

### Behavioral results

#### RTs

The two-way rm-ANOVA revealed both main effects to be significant. The main effect of the condition showed that participants were faster in the congruent trials compared to the incongruent trials (*F* (1, 88) = 373.631, *P *< 0.001, ɳ^2^ = 0.809). The main effect of the group showed that the groups significantly differed in their overall RTs (*F* (2, 88) = 11.536, *P* < 0.001, ɳ^2^ = 0.208). Additionally, we found a significant interaction between the condition and group (*F* (2, 88) = 11.229, *P* < 0.001, ɳ^2^ = 0.203).

Post hoc comparisons with a Fisher’s Least Significant Difference test revealed that the OAs were slower in both the congruent and incongruent trials than the AD and YA groups (Table 3). Their mean RTs in the congruent trials was 82 ms slower (95% CI [41, 124], *P* < 0.001) and in the incongruent trials 137 ms slower than the AD (95% CI [85, 189], *P* < 0.001). Compared to the YA, OAs were 46 ms slower in the congruent trials (95% CI [3, 89], *P* < 0.05) and 57 ms slower in the incongruent trials (95% CI [3, 111], *P* < 0.05). The difference in the performance between AD and YA was significant only in the incongruent condition, with the AD being the fastest group (AD-YA-incongruent: −80 ms, 95% CI [−132, −28], *P* < 0.01; congruent condition *P* = 0.085).

**Table 3.** Descriptive statistics

	Group		
	AD	YA	OA
Condition	*M (SD)*	*M (SD)*	*M (SD)*
Congruent *(in ms)*	526 (62)	563 (114)	608 (60)
Incongruent *(in ms)*	592 (78)	672 (133)	729 (93)
Raw interference effect *(in ms)*	66 (42)	109 (42)	120 (60)
PCE *(in %)*	10.71 (6.93)	17.81 (6.87)	19.67 (9.73)

*Note:* AD  (*n* = 33), YAs (*n* = 29), OAs (*n* = 29). Raw interference effect = RT incongruent - RT congruent.

#### PCE

There was a statistically significant difference between the three groups as determined by one-way ANOVA (*F* (2, 88) = 11.229, *P* < 0.001). A Least Significant Difference post hoc test revealed that the AD group was the one with the lowest PCE compared to the YA group (mean difference: −7.10, 95% CI [−11, −3], *P* < 0.001) and the OA group (mean difference: −8.96, 95% CI [−13, −5], *P* < 0.001). YA and OA groups did not differ with regard to the PCE (*P* = 0.372).

Multivariate linear regression analysis revealed that age was a significant predictor of the PCE (*ß* = 0.162, standard error (SE) = 0.041, *F* (1, 89) = 15.750, *P* < 0.001, ɳ^2^ = 0.150).

#### Accuracy

With regard to the percentage of correct reactions, the main effect of the Condition was significant (*F* (1, 88) = 39.381, *P* < 0.001, ɳ^2^ = 0.309), with a higher percent of correct reactions in the congruent condition. Neither the main effect of Group (*P* = 0.797) nor the interaction Condition × Group (*P* = 0.936) were significant, indicating that the three groups did not differ in accuracy across different conditions. All three groups showed a very low percentage of errors overall (Table 4).

**Table 4.** Descriptive statistics for accuracy (in % correct)

	Group		
	AD	YA	OA
Condition	*M* (SD)	*M* (SD)	*M* (SD)
Congruent accuracy	0.981 (0.029)	0.992 (0.014)	0.985 (0.019)
Incongruent accuracy	0.921 (0.085)	0.927 (0.083)	0.917 (0.134)

*Note:* AD (*n* = 33), YA (*n* = 29), OAs (*n* = 29).

### Neuroimaging results

The first goal of the group-level analysis was to validate our study by replicating the neuroimaging results from previous studies (Brass *et al.*, 2009; Darda and Ramsey, 2019) in our YA sample.

#### Manipulation check for the YAs

**Fig. 1.**
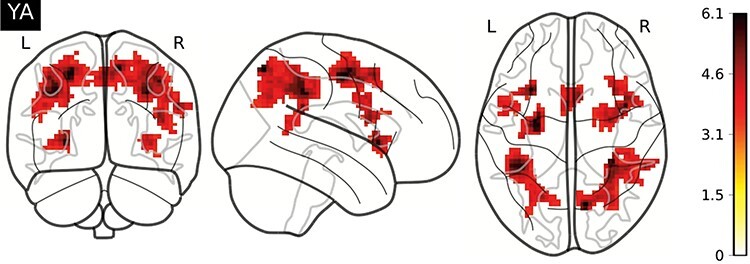
Manipulation checks of the fMRI data of the YA. Activation maps evoked by effects of interference (interference: incongruent > congruent) in YA (*n* = 29, one-sample *t*-test, initial cluster-defining threshold *P* < 0.001 uncorrected, *P* < 0.05, FWE corrected at the cluster level, with a cluster size threshold *k* = 37). We found brain activations in the right precuneus, left MFG, left IPL, right precentral gyrus, right SMA, bilateral insula, right inferior frontal and the MTG. The figure was created with nilearn.plotting.plot_glass_brain (Abraham *et al.*, 2014).

The following brain regions resulted in being significantly active from the one-sample *t*-test computed on the interference contrast (*P* < 0.05 the family-wise error rate (FWE) cluster level, cluster size *k* = 37, *P* < 0.001 initial threshold) in the YA group: right precuneus (including right temporoparietal junction and right supramarginal gyrus), left middle frontal gyrus (MFG), left IPL, right precentral gyrus, right supplementary motor area (SMA), bilateral insula, right inferior frontal and the middle temporal gyrus (MTG) (Table 5, Figure 1).

**Table 5.** Task-induced neural activation evoked by effects of interference (interference: incongruent > congruent), YA group (*n* = 29, one-sample *t*-test, initial cluster-defining threshold *P* < 0.001 uncorrected, *P* < 0.05, FWE corrected at the cluster level, with a cluster size threshold *k* = 37)

Anatomical region	Hemisphere	Cluster k	*T*	Z score	MNI coordinates x, y, z in mm
Precuneus	R	514	6.15	4.85	12, −70, 55
rSMG/WM			5.96	4.75	33, −37, 37
TPJ/rSMG			5.37	4.42	57, −40, 28
MFG	L	130	6.04	4.79	−27, −7, 52
Precentral			4.41	3.81	−36, −19, 52
Frontal superior			4.29	3.73	−27, −7, 64
IPL	L	396	5.89	4.71	−36, −40, 40
Parietal superior			4.84	4.09	−21, −64, 55
Occipital middle			4.29	3.73	−30, −67, 37
Insula	L	61	5.85	4.69	−30, 17, 1
Precentral	R	169	5.69	4.60	42, 5, 46
Precentral			4.92	4.14	30, −4, 52
Frontal superior			4.55	3.90	27, −1, 61
Precentral	R	71	4.70	4.00	48, 8, 34
SMA	R	96	5.09	4.24	6, 14, 52
Insula	R	56	3.47	3.14	42, 20, −11
IFG	L	60	4.59	3.93	−45, 5, 28
MTG	R	38	4.31	3.74	45, −52, 16

*Note:* R = right, L = left; WM = white matter; precentral = precentral gyrus. Voxels were labeled according to (Tzourio-Mazoyer, *et al.*, 2002) in MRIcron (www.mricro.com) and the neuromorphometrics atlas in SPM 12.

**Fig. 2.**
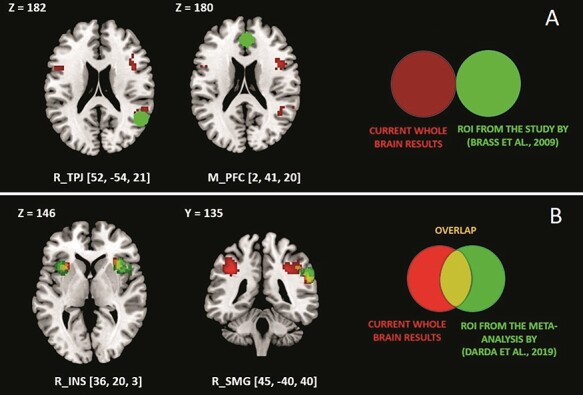
Brain regions showing increased activity for the interference contrast in the current study (in red, flexible factorial design, *P* < 0.05, FWE corrected at the cluster level) and (A) regions from the study by Brass *et al.*, (2009) (in green). The results show the cluster adjacent to the one of Brass *et al.*, (2009) in rTPJ, as indicated by the red color, and no overlap in mPFC. (B) Regions reported in the meta-analysis (in green; Darda and Ramsey, 2019). The results show an overlap in the bilateral insular and the right supramarginal gyrus, as indicated by the orange color. Coordinates are in the MNI space. The figure was created using MRIcron (www.mricro.com).

**Fig. 3.**
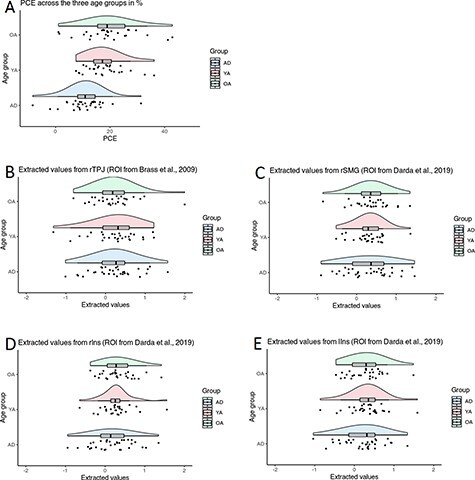
Raincloud plots of participants’ (A) PCE interference scores and extracted values from the ROIs: the right temporoparietal junction (B), right supramarginal gyrus (C), right insula (D), left insula (E) grouped by age. ADs (bottom), YAs (middle) and OAs (top). Raincloud plots represent density distribution per condition with jittered raw data, boxplots of central tendency and error. Dots are the individual scores of the participants (R package Raincloud plots, Allen *et al.*, 2019).

We could replicate other empirical findings by showing that the task used in this study activates three key areas in a similar way as previously found: rTPJ, rSMG and the bilateral Ins. The cluster we identified in rTPJ was adjacent to the one of Brass *et al.*, (2009) (Figure 2A). We also observed a partial overlap in the rSMG and a greater alignment in the bilateral Ins, resulting from the meta-analysis by Darda and Ramsey (2019) (Figure 2B; see Figure 3 for the ROI data point distribution). Contrary to the study by Brass *et al.*, (2009), we found no activation in the mPFC associated with the interference effect. This was the only ROI we could not replicate with our study design.

#### Brain–behavior correlations

No correlations between the ROIs extracted values and the PCE scores, controlling for age, were significant (rTPJ: *P* = 0.856; rSMG: *P* = 0.120, rIns, *P* = 0.053, lIns, *P* = 0.068).

#### Age-related differences in neural correlates

Results from the ROI analysis (one-way ANOVAs with the three age groups) did not show any significant age-related difference, neither for the planned ROIs by Brass *et al.*, (2009) nor for the explorative ROIs by Darda and Ramsey (2019): rTPJ (*P* = 0.993, *F* (2, 88) = 0.007, η^2^ = 0.000), rSMG (*P* = 0.899, *F* (2, 88) = 0.107, η^2^ = 0.002), rIns (*P* = 0.404, *F* (2, 88) = 0.916, η^2^ = 0.020), and lIns (*P* = 0.641, *F* (2, 88) = 0.446, η^2^ = 0.010). Correlation analyses between ROI activation and age revealed no significant results either: rTPJ: *P* = 0.870, rSMG: *P* = 0.827, rIns: *P* = 0.315 and lIns: *P* = 0.663. The multiple regression analysis aiming to associate behavioral and brain data revealed no significant findings (SM2.4 in the Supplementary material).

#### Whole-brain analysis

For the whole-brain analysis, we computed a flexible factorial design with four contrasts of interests. None of the voxels in the pairwise comparisons between the groups (AD > YA, AD < YA, OA > YA, OA < YA) showed significant differences, even when lowering the threshold to a very liberal *P* < 0.01 initial voxel selection level (*P* < 0.05 FWE at a cluster level).

## Discussion

The present study investigated behavioral and neural age-related differences in interference control in the context of a finger-lifting task initially developed to assess automatic imitation and its control. The behavioral results showed that ADs are better than YAs and OAs in interference control, reflected in their smaller interference effect, even when controlling for an overall age-related increase in RT by means of the PCE (Forbes *et al.*, 2017; Catmur and Heyes, 2011). However, when treating age as a continuous variable, age was a significant linear predictor of the PCE (as in Wermelinger *et al.*, (2018), but see SM2.1 in the Supplementary material). Most likely, the significant relationship of age on the PCE is not linear monotone but is instead driven by the group of ADs (SM2.2 in the Supplementary material), as shown by the steeper slope between AD and YA, with the curve’s slope leveling off from the YA to the OA (resembling interference control age trajectory from Bessette *et al.*, (2020)).

A possible explanation for the lack of performance differences between the YA and OA groups is that the effects of advanced age may become significant only when more complex tasks are used. For instance, it has been shown that tasks that are more automatic and do not heavily depend on executive control are less sensitive to aging (Andrés *et al.*, 2008). This explanation is also supported by the fact that the same participants as in the present study also performed a more challenging emotional egocentricity bias task, and in that case, age-related differences in both performance and neural responses were indeed observed (Riva *et al.*, 2022).

At the brain level, manipulation checks of the fMRI results in YAs revealed an overlap in the cluster adjacent to the one found by Brass *et al.*, (2009) in rTPJ but no overlap in mPFC. Second, we also detected activity in the supramarginal gyrus and in the bilateral insula, indicating that our findings for the YAs largely align with prior evidence reporting the engagement of these brain structures (Darda *et al.*, 2018; meta-analysis, Darda and Ramsey, 2019).

When addressing age-related differences at the brain level, no age differences emerged among the three groups, contradicting our expectation of different neural responses in temporoparietal and prefrontal areas. These predictions were based on behavioral and neural results (Riva *et al.*, 2016, 2022), as well as developmental neuroanatomical data (Gogtay *et al.*, 2004; Shaw *et al.*, 2008). Therefore, in our study, better behavioral performance in ADs was not associated with any matching group differences in brain activation. This, at first glance counterintuitive, mismatch between behavioral and neural data may be explained by reference to the concept of neural efficiency (Neubauer and Fink, 2009; Dunst *et al.*, 2014). This concept postulates that certain individuals use their neural resources more efficiently to achieve the same or better task performance (Neubauer and Fink, 2009; Dunst *et al.*, 2014). In other words, similar activation on the neural level might have led the ADs of our sample to a better performance on the behavioral level via a higher efficiency and behavioral relevance of the engaged networks.

On the other end of the lifespan, we had not predicted the comparable interference control performance on the behavioral level between older and younger adults, based on previous findings in the domain of emotional interference control (Riva *et al.*, 2016, confirmed regarding old *vs* young age in Riva *et al.*, 2022). Yet, some evidence aligns with our findings (see Langenecker *et al.*, 2004 and Verhaeghen, 2011 for meta-analyses; see Lustig and Jantz, 2015 for review). A meta-analysis of 176 studies could not find much support for general inhibition deficits in older age (Rey-Mermet and Gade, 2018). Another study on age-related differences in interference control using the Stroop task (Stroop, 1935) reported high within- and between-person variability, which was especially pronounced in older age, where some OAs showed similar performance to middle-aged or younger adults (Gajewski *et al.*, 2020; see also a study on cognitive reserve and aging, Cotrena *et al.*, 2021). According to the scaffolding theory by Park and Reuter-Lorenz (2009) or the CRUNCH theoretical framework (The Compensation-Related Utilization of Neural Circuits; Reuter-Lorenz and Cappell, 2008), a preserved behavioral performance in OA, associated with an extensive neural activation, reflects an attempt to compensate for a possible age-related decline. However, no such evidence was found in the present study, as we did not find any group differences in neural responses, neither in targeted ROIs nor in complementary whole-brain analyses. One possible explanation for this mismatch between the present and prior findings might be the age range from which we recruited. Based on our inclusion criteria and considering the feasibility of recruiting a sufficiently large sample of OAs for a neuroimaging study, the group of OAs was relatively younger compared to other studies on inhibitory control. For example, a study by Langenecker *et al.*, (2004) found a comparable behavioral performance in the Stroop task between YA and OA but a higher neural activity in the older sample (mean age = 71 *vs* 64 years in our sample). Thus, it might be that neural compensation mechanisms become evident only later in life.

With these interpretations notwithstanding, it might be raised that our study may not have been appropriately powered to detect putative group differences. However, while we did not perform a formal *a priori* power analysis when planning theoverarching project within which the present study was embedded (cf. Darda *et al.*, 2018), we argue, based on post hoc power considerations, that our study was sufficiently powered to detect upper medium to large effect sizes (SM1.2 in the Supplementary material).

Regarding study limitations, we have already mentioned that the version of the task we used does not allow us to control for confounds related to spatial alignment. Future studies on imitation inhibition across the lifespan should either use tasks that disentangle spatial from imitative effects (e.g. Sowden and Catmur, 2015; Sowden *et al.*, 2016) or tasks high on ecological validity (mimicry paradigm; Chartrand and Bargh, 1999; Genschow *et al.*, 2017). Notably, a recent high-powered MRI study, which employed independent functional ROIs, showed no evidence that the version of the task we used is a valid measure of covert imitative response tendencies. Therefore, the results of the present research might reflect an age-related preservation of the domain-general conflict resolution system rather than the system tied to operations within the theory-of-mind network (see the current discourse about the domain specificity of the SRC tasks: Ramsey, 2018; cf. Cracco and Brass, 2019). Second, this study was part of a larger project (Riva *et al.*, 2022), which imposed specific inclusion and exclusion criteria. For this reason, we tested only females (see Darda *et al.*, 2018, 2020 for sex differences) and did not have a middle-aged group. These specific exclusion criteria limit the generalizability of our findings. Future studies should thus investigate interference control in male individuals to assess the generalizability of the current findings to the other sex/gender; ideally, this would be done so that the full range of the lifespan is covered.

In conclusion, our study extends prior work by showing that the task we used activates a network of brain structures consistently identified by a recent meta-analysis of fMRI studies in YAs (Darda and Ramsey, 2019) and also in ADs and OAs. This network was not differentially engaged at different ages, which is suggestive of processes such as neural efficiency and the preservation of brain function in the cohorts investigated. Our study thus provides a solid foundation against which future research can compare and expand its findings on interference control as well as, when using the necessary task controls, the neural bases of automatic imitation across the lifespan.

## Supplementary Material

nsad034_Supp

## Data Availability

The data that support the findings of this study are openly available here https://osf.io/s79th/

## References

[R1] Abraham A. , Pedregosa F., Eickenberg M., et al. (2014). Machine learning for neuroimaging with scikit-learn. *Frontiers in Neuroinformatics*, 8, 14. 10.3389/fninf.2014.00014 PMC3930868 24600388

[R2] Aicken M.D. , Wilson A.D., G. J.H., Mon-Williams M. (2007). Methodological issues in measures of imitative reaction times. *Brain and Cognition*, 63(3), 304–8. 17070640 10.1016/j.bandc.2006.09.005

[R3] Allen M. , Poggiali D., Whitaker K., Marshall T.R., Kievit R.A. (2019). Raincloud plots: a multi-platform tool for robust data visualization. *Wellcome Open Research*, 4, 63. 10.12688/wellcomeopenres.15191.1 PMC6480976 31069261

[R4] Andrés P. , Guerrini C., Phillips L.H., Perfect T.J. (2008). Differential effects of aging on executive and automatic inhibition. *Developmental Neuropsychology*, 33(2), 101–23. 18443972 10.1080/87565640701884212

[R5] Babel M. (2011). Imitation in speech. *Acoustics Today*, 7(4), 16–22.

[R6] Bedard A.-C. , Nichols S., Barbosa J.A., Schachar R., Logan G.D., Tannock R. (2002). The development of selective inhibitory control across the life span. *Developmental Neuropsychology*, 21(1), 93–111. 12058837 10.1207/S15326942DN2101_5

[R7] Bessette K.L. , Karstens A.J., Crane N.A., et al. (2020). A lifespan model of interference resolution and inhibitory control: risk for depression and changes with illness progression. *Neuropsychology Review*, 30(4), 477–98. 31942706 10.1007/s11065-019-09424-5 PMC7363517

[R8] Bischoff C. , Reutner L., and Hansen J. (2020). The snacking chameleon: psychological proximity increases imitation of food intake independently of brand choice. *Foods*, 9(2), 228. 10.3390/foods9020228 PMC7074025 32098066

[R9] Blakemore S.-J. (2008). The social brain in adolescence. *Nature Reviews Neuroscience*, 9(4), 267–77. 18354399 10.1038/nrn2353

[R10] Blakemore S.-J. (2012). Development of the social brain in adolescence. *Journal of the Royal Society of Medicine*, 105(3), 111–6. 22434810 10.1258/jrsm.2011.110221 PMC3308644

[R11] Botvinik-Nezer R. , Holzmeister F., Camerer C.F., et al. (2020). Variability in the analysis of a single neuroimaging dataset by many teams. *Nature*, 582(7810), 84–8. 32483374 10.1038/s41586-020-2314-9 PMC7771346

[R12] Brass M. , Bekkering H., Wohlschläger A., Prinz W. (2000). Compatibility between observed and executed finger movements: comparing symbolic, spatial, and imitative cues. *Brain and Cognition*, 44(2), 124–43. 11041986 10.1006/brcg.2000.1225

[R13] Brass M. , Derrfuss J., von Cramon D.Y. (2005). The inhibition of imitative and overlearned responses: a functional double dissociation. *Neuropsychologia*, 43(1), 89–98. 15488909 10.1016/j.neuropsychologia.2004.06.018

[R14] Brass M. , Ruby P., Spengler S. (2009). Inhibition of imitative behaviour and social cognition. *Philosophical Transactions of the Royal Society B: Biological Sciences*, 364(1528), 2359–67. 10.1098/rstb.2009.0066 PMC2865080 19620107

[R15] Catmur C. , Heyes C. (2011). Time course analyses confirm independence of imitative and spatial compatibility. *Journal of Experimental Psychology. Human Perception and Performance*, 37(2), 409–21. 20731523 10.1037/a0019325

[R16] Chartrand T.L. , and Bargh J.A. (1999). The chameleon effect: the perception–behavior link and social interaction. *Journal of Personality and Social Psychology*, 76(6), 893–910. 10402679 10.1037//0022-3514.76.6.893

[R17] Chen Y.-C. , Chen-C.-C., Decety J., Cheng Y. (2014). Aging is associated with changes in the neural circuits underlying empathy. *Neurobiology of Aging*, 35(4), 827–36. 24211010 10.1016/j.neurobiolaging.2013.10.080

[R18] Christiner M. , Reiterer S.M. (2013). Song and speech: examining the link between singing talent and speech imitation ability. *Frontiers in Psychology*, 4, 874. 10.3389/fpsyg.2013.00874 PMC3837232 24319438

[R19] Cotrena C. , Branco L.D., Ponsoni A., Shansis F.M., Fonseca R.P. (2021). Cognitive reserve may outperform age, mood and psychiatric comorbidities as a predictor of executive functioning in bipolar disorder and healthy adults. *Journal of Clinical and Experimental Neuropsychology*, 43(6), 611–22. 34730064 10.1080/13803395.2021.1981251

[R20] Cracco E. , Bardi L., Desmet C., et al. (2018). Automatic imitation: a meta-analysis. *Psychological Bulletin*, 144(5), 453–500. 29517262 10.1037/bul0000143

[R21] Cracco E. , Brass M. (2019). Reaction time indices of automatic imitation measure imitative response tendencies. *Consciousness and Cognition*, 68, 115–8. 30638700 10.1016/j.concog.2019.01.001

[R22] Cross K.A. , Iacoboni M. (2014). To imitate or not: avoiding imitation involves preparatory inhibition of motor resonance. *NeuroImage*, 91, 228–36. 24473096 10.1016/j.neuroimage.2014.01.027 PMC4117687

[R23] Czekóová K. , Shaw D.J., Lamoš M., Špiláková B., Salazar M., Brázdil M. (2021). Imitation or polarity correspondence? Behavioural and neurophysiological evidence for the confounding influence of orthogonal spatial compatibility on measures of automatic imitation. *Cognitive, Affective & Behavioral Neuroscience*, 21(1), 212–30. 10.3758/s13415-020-00860-y PMC7994238 33432546

[R24] Darda K.M. , Butler E.E., Ramsey R. (2018). Functional specificity and sex differences in the neural circuits supporting the inhibition of automatic imitation. *Journal of Cognitive Neuroscience*, 30(6), 914–33. 29561236 10.1162/jocn_a_01261

[R25] Darda K.M. , Butler E.E., Ramsey R. (2020). Individual differences in social and non-social cognitive control. *Cognition*, 202, 104317. 10.1016/j.cognition.2020.104317 32460970

[R26] Darda K.M. , Ramsey R. (2019). The inhibition of automatic imitation: a meta-analysis and synthesis of fMRI studies. *NeuroImage*, 197, 320–9. 31028924 10.1016/j.neuroimage.2019.04.059

[R27] Decety J. , Lamm C. (2007). The role of the right temporoparietal junction in social interaction: how low-level computational processes contribute to meta-cognition. *The Neuroscientist*, 13(6), 580–93. 17911216 10.1177/1073858407304654

[R28] Dunst B. , Benedek M., Jauk E., et al. (2014). Neural efficiency as a function of task demands. *Intelligence*, 42, 22–30. 24489416 10.1016/j.intell.2013.09.005 PMC3907682

[R29] Eickhoff S.B. , Stephan K.E., Mohlberg H., et al. (2005). A new SPM toolbox for combining probabilistic cytoarchitectonic maps and functional imaging data. *NeuroImage*, 25(4), 1325–35. 15850749 10.1016/j.neuroimage.2004.12.034

[R30] Ferguson H.J. , Brunsdon V.E.A., Bradford E.E.F. (2021). The developmental trajectories of executive function from adolescence to old age. *Scientific Reports*, 11(1), 1382. 10.1038/s41598-020-80866-1 PMC7809200 33446798

[R31] Forbes P.A. , Wang Y., de C Hamilton A.F. (2017). STORMy interactions: gaze and the modulation of mimicry in adults on the autism spectrum. *Psychonomic Bulletin & Review*, 24(2), 529–35. 27506527 10.3758/s13423-016-1136-0 PMC5389998

[R32] Frangou S. , Modabbernia A., Williams S.C.R., et al. (2022). Cortical thickness across the lifespan: data from 17,075 healthy individuals aged 3–90 years. *Human Brain Mapping*, 43(1), 431–51. 33595143 10.1002/hbm.25364 PMC8675431

[R33] Friston K.J. , Frith C.D., Frackowiak R.S., Turner R. (1995). Characterizing dynamic brain responses with fMRI: a multivariate approach. *NeuroImage*, 2(2), 166–72. 9343599 10.1006/nimg.1995.1019

[R34] Gajewski P.D. , Falkenstein M., Thönes S., Wascher E. (2020). Stroop task performance across the lifespan: high cognitive reserve in older age is associated with enhanced proactive and reactive interference control. *NeuroImage*, 207, 116430. 10.1016/j.neuroimage.2019.116430 31805383

[R35] Gallagher H.L. , Frith C.D. (2003). Functional imaging of ‘theory of mind. *Trends in Cognitive Sciences*, 7(2), 77–83. 12584026 10.1016/s1364-6613(02)00025-6

[R36] Genschow O. , Hansen J., Wänke M., Trope Y. (2019). Psychological distance modulates goal-based versus movement-based imitation. *Journal of Experimental Psychology. Human Perception and Performance*, 45(8), 1031–48. 31135170 10.1037/xhp0000654

[R37] Genschow O. , van Den Bossche S., Cracco E., Bardi L., Rigoni D., Brass M. (2017). Mimicry and automatic imitation are not correlated. *PLoS One*, 12(9), e0183784. 10.1371/journal.pone.0183784 PMC5587324 28877197

[R38] Gogtay N. , Giedd J.N., Lusk L., et al. (2004). Dynamic mapping of human cortical development during childhood through early adulthood. *Proceedings of the National Academy of Sciences*, 101(21), 8174–9. 10.1073/pnas.0402680101 PMC419576 15148381

[R39] Hasher L. , Zacks R.T. (1988). Working memory, comprehension, and aging: a review and a new view. *Psychology of Learning and Motivation*, 22, 193–225.

[R40] Heyes C. (2011). Automatic imitation. *Psychological Bulletin*, 137(3), 463–83. 21280938 10.1037/a0022288

[R41] Kessler J. , Markowitsch H., Denzler P. (2000). Mini-mental-status-test (MMST). *Göttingen: Beltz Test GMBH*, 24.

[R42] Klapper A. , Ramsey R., Wigboldus D., Cross E.S. (2014). The control of automatic imitation based on bottom–up and top–down cues to animacy: insights from brain and behavior. *Journal of Cognitive Neuroscience*, 26(11), 2503–13. 24742157 10.1162/jocn_a_00651

[R43] Kukull W. , Larson E., Teri L., Bowen J., McCormick W., Pfanschmidt M. (1994). The mini-mental state examination score and the clinical diagnosis of dementia. *Journal of Clinical Epidemiology*, 47(9), 1061–7. 7730909 10.1016/0895-4356(94)90122-8

[R44] Kupis L. , Goodman Z.T., Kornfeld S., et al. (2021). Brain dynamics underlying cognitive flexibility across the lifespan. *Cerebral Cortex*, 31(11), 5263–74. 34145442 10.1093/cercor/bhab156 PMC8491685

[R45] Lamm C. , Silani G., Singer T. (2015). Distinct neural networks underlying empathy for pleasant and unpleasant touch. *Cortex*, 70, 79–89. 25725510 10.1016/j.cortex.2015.01.021

[R46] Langenecker S.A. , Nielson K.A., Rao S.M. (2004). FMRI of healthy older adults during Stroop interference. *NeuroImage*, 21(1), 192–200. 14741656 10.1016/j.neuroimage.2003.08.027

[R47] Larsen B. , Luna B. (2018). Adolescence as a neurobiological critical period for the development of higher-order cognition. *Neuroscience and Biobehavioral Reviews*, 94, 179–95. 30201220 10.1016/j.neubiorev.2018.09.005 PMC6526538

[R48] Liepelt R. , Brass M. (2010). Automatic imitation of physically impossible movements. *Social Cognition*, 28(1), 59–73.

[R49] Lustig C. , Jantz T. (2015). Questions of age differences in interference control: when and how, not if? *Brain Research*, 1612, 59–69. 25451086 10.1016/j.brainres.2014.10.024

[R50] Mayas J. , Fuentes L., Ballesteros S. (2012). Stroop interference and negative priming (NP) suppression in normal aging. *Archives of Gerontology and Geriatrics*, 54(2), 333–8. 21215468 10.1016/j.archger.2010.12.012

[R51] Moran J.M. (2013). Lifespan development: the effects of typical aging on theory of mind. *Behavioural Brain Research*, 237, 32–40. 23000532 10.1016/j.bbr.2012.09.020

[R52] Natu V.S. , Gomez J., Barnett M., et al. (2019). Apparent thinning of human visual cortex during childhood is associated with myelination. *Proceedings of the National Academy of Sciences*, 116(41), 20750–9. 10.1073/pnas.1904931116 PMC6789966 31548375

[R53] Neubauer A.C. , Fink A. (2009). Intelligence and neural efficiency. *Neuroscience and Biobehavioral Reviews*, 33(7), 1004–23. 19580915 10.1016/j.neubiorev.2009.04.001

[R54] Nichols T. , and Wilke M. (2012). CorrClusTh.m (1.3) [Matlab, SPM], University of Warwick & University of Tübingen. Available: https://warwick.ac.uk/fac/sci/statistics/staff/academic-research/nichols/scripts/spm/ [June 16, 2023].

[R55] Oldfield R.C. (1971). The assessment and analysis of handedness: the Edinburgh inventory. *Neuropsychologia*, 9(1), 97–113. 5146491 10.1016/0028-3932(71)90067-4

[R56] Park D.C. , and Reuter-Lorenz P. (2009). The adaptive brain: aging and neurocognitive scaffolding. *Annual Review of Psychology*, 60, 173–96. 10.1146/annurev.psych.59.103006.093656 PMC3359129 19035823

[R57] Ramsey R. (2018). What are reaction time indices of automatic imitation measuring? *Consciousness and Cognition*, 65, 240–54. 30219745 10.1016/j.concog.2018.08.006

[R58] Range F. , Huber L., Heyes C. (2011). Automatic imitation in dogs. *Proceedings of the Royal Society B: Biological Sciences*, 278(1703), 211–7. 10.1098/rspb.2010.1142 PMC3013390 20667875

[R59] Rauchbauer B. , Grosbras M.-H. (2020). Developmental trajectory of interpersonal motor alignment: positive social effects and link to social cognition. *Neuroscience and Biobehavioral Reviews*, 118, 411–25. 32783968 10.1016/j.neubiorev.2020.07.032 PMC7415214

[R60] Reuter-Lorenz P.A. , Cappell K.A. (2008). Neurocognitive aging and the compensation hypothesis. *Current Directions in Psychological Science*, 17(3), 177–82.

[R61] Rey-Mermet A. , Gade M. (2018). Inhibition in aging: what is preserved? What declines? A meta-analysis. *Psychonomic Bulletin & Review*, 25(5), 1695–716. 29019064 10.3758/s13423-017-1384-7

[R62] Riva F. , Lenger M., Kronbichler M., Lamm C., Silani G. (2022). The role of right supra-marginal gyrus and secondary somatosensory cortex in age-related differences in human emotional egocentricity. *Neurobiology of Aging*, 112, 102–10. 35104721 10.1016/j.neurobiolaging.2022.01.002

[R63] Riva F. , Triscoli C., Lamm C., Carnaghi A., Silani G. (2016). Emotional egocentricity bias across the life-span. *Frontiers in Aging Neuroscience*, 8, 74. 10.3389/fnagi.2016.00074 PMC4844617 27199731

[R64] Riva F. , Tschernegg M., Chiesa P.A., et al. (2018). Age-related differences in the neural correlates of empathy for pleasant and unpleasant touch in a female sample. *Neurobiology of Aging*, 65, 7–17. 29407469 10.1016/j.neurobiolaging.2017.12.028

[R65] Scheflen A.E. (1964). The significance of posture in communication systems. *Psychiatry*, 27(4), 316–31. 14216879 10.1080/00332747.1964.11023403

[R66] Schurz M. , Radua J., Tholen M.G., et al. (2020). Toward a hierarchical model of social cognition: a neuroimaging meta-analysis and integrative review of empathy and theory of mind. *Psychological Bulletin*, 147(3), 293–327. 33151703 10.1037/bul0000303

[R67] Schurz M. , Tholen M.G., Perner J., Mars R.B., Sallet J. (2017). Specifying the brain anatomy underlying temporo‐parietal junction activations for theory of mind: a review using probabilistic atlases from different imaging modalities. *Human Brain Mapping*, 38(9), 4788–805. 28608647 10.1002/hbm.23675 PMC6867045

[R68] Schuwerk T. , Schurz M., Müller F., Rupprecht R., Sommer M. (2017). The rTPJ’s overarching cognitive function in networks for attention and theory of mind. *Social Cognitive and Affective Neuroscience*, 12(1), 157–68. 27798260 10.1093/scan/nsw163 PMC5390694

[R69] Shaw P. , Kabani N.J., Lerch J.P., et al. (2008). Neurodevelopmental trajectories of the human cerebral cortex. *Journal of Neuroscience*, 28(14), 3586–94. 18385317 10.1523/JNEUROSCI.5309-07.2008 PMC6671079

[R70] Silani G. , Lamm C., Ruff C.C., Singer T. (2013). Right supramarginal gyrus is crucial to overcome emotional egocentricity bias in social judgments. *The Journal of Neuroscience*, 33(39), 15466–76. 24068815 10.1523/JNEUROSCI.1488-13.2013 PMC6618458

[R71] Silberzahn R. , Uhlmann E.L., Martin D.P., et al. (2018). Many analysts, one data set: making transparent how variations in analytic choices affect results. *Advances in Methods and Practices in Psychological Science*, 1(3), 337–56.

[R72] Sowden S. , Catmur C. (2015). The role of the right temporoparietal junction in the control of imitation. *Cerebral Cortex*, 25(4), 1107–13. 24177989 10.1093/cercor/bht306 PMC4380005

[R73] Sowden S. , Koehne S., Catmur C., Dziobek I., Bird G. (2016). Intact automatic imitation and typical spatial compatibility in autism spectrum disorder: challenging the broken mirror theory: intact automatic imitation in autism. *Autism Research*, 9(2), 292–300. 26112060 10.1002/aur.1511

[R74] Spengler S. , von Cramon D.Y., Brass M. (2009). Control of shared representations relies on key processes involved in mental state attribution. *Human Brain Mapping*, 30(11), 3704–18. 19517530 10.1002/hbm.20800 PMC6870802

[R75] Steinbeis N. (2016). The role of self–other distinction in understanding others’ mental and emotional states: neurocognitive mechanisms in children and adults. *Philosophical Transactions of the Royal Society B: Biological Sciences*, 371(1686), 20150074. 10.1098/rstb.2015.0074 PMC4685520 26644593

[R76] Stroop J.R. (1935). Studies of interference in serial verbal reactions. *Journal of Experimental Psychology*, 18(6), 643–62.

[R77] Stürmer B. , Aschersleben G., Prinz W. (2000). Correspondence effects with manual gestures and postures: A study of imitation. *Journal of Experimental Psychology. Human Perception and Performance*, 26(6), 1746–59. 11129371 10.1037//0096-1523.26.6.1746

[R78] Tzourio-Mazoyer N. , Landeau B., Papathanassiou D., et al. (2002) Automated Anatomical Labeling of Activations in SPM Using a Macroscopic Anatomical Parcellation of the MNI MRI Single-Subject Brain. *NeuroImage*, 15, 273–89. 11771995 10.1006/nimg.2001.0978

[R79] Verhaeghen P. (2011). Aging and executive control: reports of a demise greatly exaggerated. *Current Directions in Psychological Science*, 20(3), 174–80. 25866452 10.1177/0963721411408772 PMC4389903

[R80] Virhia J. , Kotz S.A., Adank P. (2019). Emotional state dependence facilitates automatic imitation of visual speech. *Quarterly Journal of Experimental Psychology*, 72(12), 2833–47. 10.1177/1747021819867856 31331238

[R81] Wermelinger S. , Gampe A., Behr J., Daum M.M. (2018). Interference of action perception on action production increases across the adult life span. *Experimental Brain Research*, 236(2), 577–86. 29249051 10.1007/s00221-017-5157-3

