# Supplementary material for: Age-related differences in interference control in the context of a finger-lifting task: an fMRI study
Source: Soc Cogn Affect Neurosci. 2023 Jun 6;18(1):nsad034. doi: 10.1093/scan/nsad034 (PMC10329405; doi:10.1093/scan/nsad034)
Supplement: nsad034_Supp [file nsad034_supp.zip › scan-22-109-File002.docx]

## Age-related differences in interference control in the context of a finger-lifting task: an fMRI study

**SUPPLEMENTARY MATERIAL**

Federica Riva^*1^, Ekaterina Pronizius^*1^, Melanie Lenger^2^, Martin Kronbichler^2,3^, Giorgia Silani^4†^, Claus Lamm^1†^

*^1^Social, Cognitive and Affective Neuroscience Unit, Department of Cognition, Emotion, and Methods in Psychology, Faculty of Psychology, University of Vienna, Vienna, Austria*

*^2^Centre for Cognitive Neuroscience, University of Salzburg, Kapitelgasse 4-6, 5020 Salzburg, Austria;*

*^3^Neuroscience Institute, Christian Doppler Clinic, Paracelsus Medical University, Strubergasse 21 5020 Salzburg, Austria;*

*^4^Department of Clinical and Health Psychology, Faculty of Psychology, University of Vienna, Vienna, Austria;*

****Shared authorship.*** FR and EP contributed equally to this manuscript.

^†^***Corresponding and shared senior authors:***

Giorgia Silani, Claus Lamm

Faculty of Psychology, University of Vienna

*Address:* Liebiggasse 5, 1010 Vienna, Austria
*E-mail:* [giorgia.silani@univie.ac.at](mailto:giorgia.silani@univie.ac.at), [claus.lamm@univie.ac.at](mailto:claus.lamm@univie.ac.at)

## SM1. Materials and Methods

### SM1.1 Procedure

At the beginning of the session, participants filled in the MRI safety questionnaire and signed the informed consent. For adolescents, we also obtained written informed *parental* consent. Then, the experimenters explained the structure of the sessions and the different tasks and introduced the participants to the confederates needed for the other two tasks. Subsequently, the participants were accompanied in the scanner and performed the three tasks. The empathy and the EEB tasks were always performed one after the other, while the finger-lifting task was randomly administered before or after the other two tasks. After the MRI session, participants filled out questionnaires and were debriefed. In total, the whole session lasted for about 60 minutes. The paradigms and the results of the empathy and the EEB task are not the focus of the present paper but are documented elsewhere (Riva et al., 2018, 2022).

### SM1.2 Post-hoc power considerations

While post-hoc power analyses based on collected data can be misleading (Zhang et al., 2019), we tried to estimate for which effect sizes our study would have been sufficiently powered. To this end, we calculated the required sample sizes for a power of 0.8 and 0.05 alpha error probability with assumed “standard” low (0.1), medium (0.25), and high (0.4) effect sizes using G*Power (Erdfelder et al., 1996). This revealed required sample sizes of 969, 159, and 66 and that our sample size of N = 90 would be required for an effect size around 0.33, i.e., between upper medium and large. We thus conclude that our study might have lacked sufficient power to detect small to (lower) medium effect sizes, but was appropriately powered to detect high and upper medium effects sizes for the behavioral data (one analysis) and for large effect sizes for the neuroimaging data (due to repeated testing requiring family-wise error adjustments and thus reducing power).

### SM1.3 Exclusion of the factor finger from the rm-ANOVAs

For RTs, we found a significant main effect of *Finger* (*F* (1, 88) = 16.301, *p* < .001, ɳ^2^ = .156) and a significant *Finger x Condition* interaction (*F* (1, 88) = 8.694, *p* < .01, ɳ^2^ = .090), with the middle finger being associated with faster reaction times, especially in the incongruent condition. Yet, the absence of a two- and three-way interaction with age group (indicating that this difference between finger movements was of similar size in all age groups) showed that this difference seemed of low relevance for the main analyses (*Finger x Group* *p* = .727 and *Finger x Group x Condition p = .311*). For accuracy as the dependent variable, neither was the main effect of the *Finger* significant, nor its interaction with the group or condition (all *p*s > .153). For simplicity of the models and analyses, we thus decided to pool across fingers in the analyses reported in the paper.

### SM1.4 fMRI analysis: regressors and timing

Four task regressors and six regressors of no interest were included in the model and convolved with SPM’s canonical hemodynamic response function. The four task regressors modeled the following segments of the task: first, the period in which a picture of the still hand was presented at the beginning of a trial (irrespective of whether the upcoming finger-to-lift was congruent or incongruent to the one supposed to be executed by the participant); second, the period in which the go command and the concomitant lifting finger was shown for correct congruent trials; third, the same as the second but for incongruent trials, and fourth, a regressor including all error trials. The regressors were treated as in an event-related design, so the duration was set to 0. Since we were not interested in the differences between the two fingers, index and middle finger trials were considered together. The six nuisance regressors of no interest representing realignment parameters were included to correct for residual effects of head motion.

### SM1.5 ROIs definition and construction

In the study by Brass et al. (2009), the authors identified two brain regions (mPFC and rTPJ) that were significantly involved in the inhibition control. Based on the reported coordinates, we built two 9-mm radius spheres, which were subsequentially used in the ROI analysis. Before that, we transformed the reported TAL peak coordinates into the MNI space^[[1]](#footnote-1)^: TPJ TAL (52, -54, 21) and its corresponding MNI coordinates (52, -56, 20); mPFC TAL (1, 39, 18) and its corresponding MNI coordinates (2, 41, 20).

In the meta-analysis (Darda & Ramsey, 2019), the authors computed separate meta-analyses for studies: a) where the imitative and the spatial compatibility effects were distinguishable; b) where the two effects were not distinguishable, providing ROIs for the general compatibility. Since, in our study, imitative and spatial effects in the task are not separable, we used the ROIs resulting from the general compatibility effect. These brain regions include the right posterior supramarginal gyrus (rSMG), the right Insula (rIns), and the left Insula (lIns). The ROIs from the meta-analysis (Darda & Ramsey, 2019) are available on neurovault (*Activation_FWE_extent_stringent* <https://neurovault.org/collections/5377/>). Mean region activity was extracted for each subject for each region with REX, an SPM toolbox (<https://www.nitrc.org/projects/rex/>).

## SM2. Results & Discussion

### SM2.1 Results of the multivariate linear regression

Age (continuous) significantly predicted PCE interference effect (*ß* = .162, SE = .041, F (1, 89) = 15.750, p < .001, ɳ2 = .150), congruent RTs (*ß* = 1.588, SE = .411, *F* (1, 89) = 14.906, *p* < .001, ɳ^2^ =.143) and incongruent RTs (*ß* = 2.580, SE = .523, *F* (1, 89) = 24.314, *p* < .001, ɳ^2^ =.215), as estimated with multivariate linear regression analysis. In the analysis, where age was a categorical variable, OA were slower in both, the congruent and incongruent trials compared to the AD and YA groups. The difference in the performance between AD and YA was significant only in the incongruent condition, with the AD being the fastest group.

The results of the multivariate linear regression on incongruent RTs are comparable to the study by Wermelinger et al. (2018), where age was a significant predictor, with the older adults being the slowest group. We explain these results in terms of the general deterioration of executive functions caused by age-related physiological processes (Gottsdanker, 1982). Previous empirical studies show that aging has a heterogeneous effect on different components of interference control (Korsch et al., 2014), and simple reaction time responses increase with age (Gottsdanker, 1982).

Contrarily to the study by Wermelinger et al. (2018), in our study, age was a significant linear predictor of the reaction times in the congruent trials as well, although to a lesser magnitude. Yet, a group comparison revealed no difference between adolescents and young adults. Most likely, the significant result of the linear regression was driven by the group of older adults, and the relationship between age and congruent RT is not linear monotone. Taken together, these findings provide additional support that aging has a heterogenous effect on the perception of incongruent as compared to congruent actions.

While comparing the present results to the results of Wermelinger et al. (2018), we have to acknowledge the methodological differences between the two studies. Our study expanded age-related effects into a much younger group as in the study of Wermelinger et al. (2018; min. age = 20 in their study, min. age = 14 in the present study). Yet, we did not have a middle-aged group. Secondly, the study by Wermelinger et al. (2018) used baseline corrected reaction times, whereas we used an interference effect controlled for an overall reaction time differences (PCE). Third, in their study, the motor interference effect was controlled for visual input via an additional condition, which was not the case in the present study. Thus, the results may not necessarily be inconsistent but could be related to these methodological differences.

### SM2.2 Curve estimation

Age significantly predicted the PCE, as estimated with the linear regression analyses. In the analysis, where age was a categorical variable, AD was the group with the lowest PCE. The YA and OA groups did not differ regarding their PCE scores. The strength of the regression analysis is that it predicts the persistence of the relationships outside the data range. On the other hand, its shortcoming is an assumption of a monotone linear relationship between the predictor and the outcome, whereas, in real life, this relationship may be more complex. To account for the discrepancies between the results of the analyses with age as a categorical vs. a continuous variable (see above), we run a curve estimation analysis.

The results of the curve estimation procedure with age as a predictor and the PCE score as an outcome revealed that the inverse^[[2]](#footnote-2)^ curve of the regression model has the best fit in terms of its coefficients (e.g., higher F-value and R Square). In our study, the inverse relationship of age on the PCE score corresponds to a steeper increase from the AD to the YA, with the curve leveling off from the YA to the OA. These results are in line with the results of the group comparison (AD < YA = OA), with age being a categorical variable.

As seen in *Table 1*, the fit of the linear regression model is comparable to that of the other models. For reasons of simplicity and to make it more comparable to the analysis of Wermelinger et al. (2018), we adopted the linear trend in the main part of the manuscript. We acknowledge, however, that the linear trend is probably driven by the AD. When excluding the AD from the linear regression analysis, the relationship of age on the PCE is no longer significant (*p* = .20).

Table 1

*Curve estimation. Model Summary and Parameter Estimates*

|  | Model Summary | | | | |  | Parameter Estimates | | | |
| --- | --- | --- | --- | --- | --- | --- | --- | --- | --- | --- |
| Equation | R Square | F | df1 | df2 | Sig. |  | Constant | b1 | b2 | b3 |
| Linear | .150 | 15.750 | 1 | 89 | .000 |  | 10.373 | .162 |  |  |
| Logarithmic | .169 | 18.140 | 1 | 89 | .000 |  | -4.421 | 6.072 |  |  |
| **Inverse** | **.189** | **20.710** | **1** | **89** | **.000** |  | **23.577** | **-186.577** |  |  |
| Quadratic | .157 | 8.173 | 2 | 88 | .001 |  | 6.273 | .443 | -.003 |  |
| Cubic | .288 | 11.746 | 3 | 87 | .000 |  | -24.894 | 3.529 | -.090 | .001 |

*Note:* Dependent variable = PCE (percentage congruency effect); independent variable = age. The inverse model equation: Y = b0 + (b1/t).

### SM2.3 Manipulation checks of the fMRI data of the whole sample

For the second-level analysis, we used a flexible factorial design with one between-subjects factor *group* (adolescents, young adults, older adults) and one within-subject factor *interference* (incongruent - congruent). As in the group of YA, on the whole group level, our findings align with prior evidence, indicating that the task as used here engaged neural processing compatible with interference control. More importantly, since we do not find any group differences on the neural level, we conclude that the task activates neural regions involved in the interference control *independently of age.*

On the whole group level, we found an overlap in the cluster adjacent to the one of Brass et al. (2009) in rTPJ, activity in the supramarginal gyrus, and in the bilateral insula. Similar to the manipulation check in YA, we found no activity in mPFC, which was reported by Brass et al. (2009).


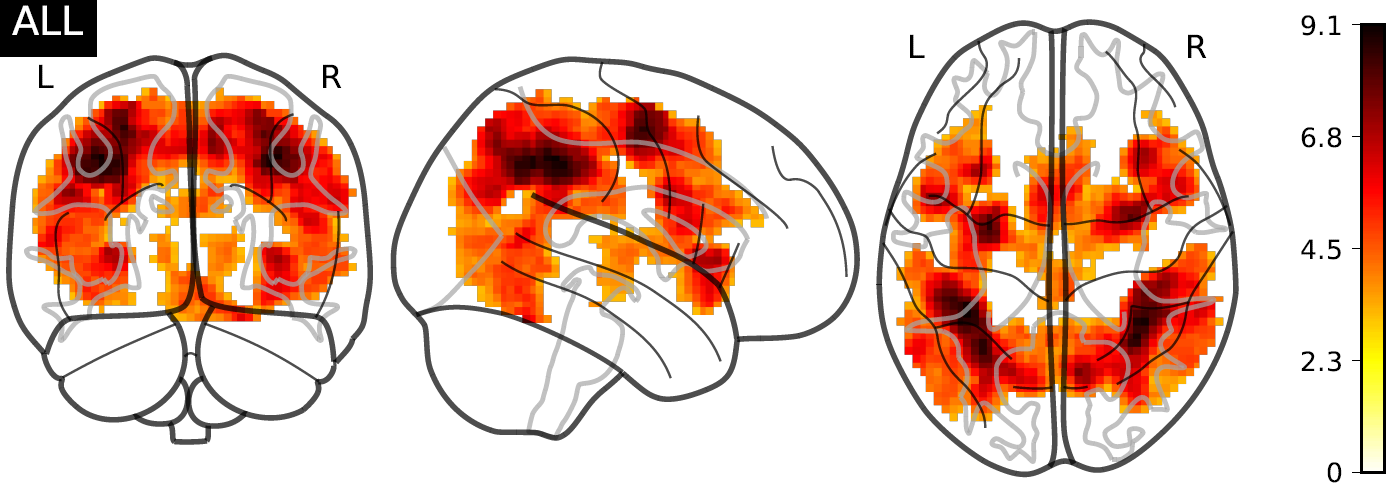


*Figure 1.* Manipulation checks of the fMRI data of the whole sample. Activation maps evoked by the interference effect across all groups (N = 91, initial cluster-defining threshold p < .001 uncorrected, p < .05, FWE corrected at the cluster level, with a cluster size threshold k = 37). The glass brain view highlights activations, amongst others, in key areas identified in previous work on automatic imitation inhibition, including the temporoparietal junction (incl. supramarginal gyrus), bilateral insular cortex, and various subparts of the parietal cortices. However, no activation was found in the medial prefrontal cortex (see main text for further discussion). The figure was created with nilearn.plotting.plot_glass_brain (Abraham et al., 2014).

### SM2.4 Multiple regression

We specified a multiple regression model in SPM with the fMRI interference contrasts for every participant as the dependent variable. In this model, the PCE behavioral score was included as a covariate on the individual level.

None of the voxels survived the significance threshold. Lowering the threshold to a peak level (uncorrected) resulted in a cluster in the right inferior temporal gyrus (66,-19,-20; *k* = 7, *T* = 4.40, *Z* = 4.18) for the Interference- contrast and in white matter for the Interference+ contrast (21,-16,52; *k* = 16, *T* = 4.20, *Z* = 4.00). None of these activations, apart from not surviving strict statistical criteria, should thus be considered meaningful.

**References**

Abraham, A., Pedregosa, F., Eickenberg, M., Gervais, P., Mueller, A., Kossaifi, J., Gramfort, A., Thirion, B., & Varoquaux, G. (2014). Machine learning for neuroimaging with scikit-learn. *Frontiers in Neuroinformatics*, *8*, 14. https://doi.org/10.3389/fninf.2014.00014

Brass, M., Ruby, P., & Spengler, S. (2009). Inhibition of imitative behaviour and social cognition. *Philosophical Transactions of the Royal Society B: Biological Sciences*, *364*(1528), 2359–2367. https://doi.org/10.1098/rstb.2009.0066

Darda, K. M., & Ramsey, R. (2019). The inhibition of automatic imitation: A meta-analysis and synthesis of fMRI studies. *NeuroImage*, *197*, 320–329. https://doi.org/10.1016/j.neuroimage.2019.04.059

Erdfelder, E., Faul, F., & Buchner, A. (1996). GPOWER: A general power analysis program. *Behavior Research Methods, Instruments, & Computers*, *28*(1), 1–11. https://doi.org/10.3758/BF03203630

Gottsdanker, R. (1982). Age and simple reaction time. *Journal of Gerontology*, *37*(3), 342–348. https://doi.org/10.1093/geronj/37.3.342

Korsch, M., Früholz, S., & Herrmann, M. (2014). Ageing differentially affects neural processing of different conflict typesâ€”an fMRI study. *Frontiers in Aging Neuroscience*, *6*. https://doi.org/10.3389/fnagi.2014.00057

Riva, F., Lenger, M., Kronbichler, M., Lamm, C., & Silani, G. (2022). The role of right supra-marginal gyrus and secondary somatosensory cortex in age-related differences in human emotional egocentricity. *Neurobiology of Aging*, *112*, 102–110. https://doi.org/10.1016/j.neurobiolaging.2022.01.002

Riva, F., Tschernegg, M., Chiesa, P. A., Wagner, I. C., Kronbichler, M., Lamm, C., & Silani, G. (2018). Age-related differences in the neural correlates of empathy for pleasant and unpleasant touch in a female sample. *Neurobiology of Aging*, *65*, 7–17. https://doi.org/10.1016/j.neurobiolaging.2017.12.028

Wermelinger, S., Gampe, A., Behr, J., & Daum, M. M. (2018). Interference of action perception on action production increases across the adult life span. *Experimental Brain Research*, *236*(2), 577–586. https://doi.org/10.1007/s00221-017-5157-3

Zhang, Y., Hedo, R., Rivera, A., Rull, R., Richardson, S., & Tu, X. M. (2019). Post hoc power analysis: Is it an informative and meaningful analysis? *General Psychiatry*, *32*(4), e100069. https://doi.org/10.1136/gpsych-2019-100069

1. <https://bioimagesuiteweb.github.io/webapp/mni2tal.html> [↑](#footnote-ref-1)
2. the inverse model equation: $Y = b0 + (b1 / t)$. [↑](#footnote-ref-2)
